# Clinical Impact of Nutritional Status and Sarcopenia in Pediatric Patients with Bone and Soft Tissue Sarcomas: A Pilot Retrospective Study (SarcoPed)

**DOI:** 10.3390/nu14020383

**Published:** 2022-01-17

**Authors:** Alberto Romano, Silvia Triarico, Emanuele Rinninella, Luigi Natale, Maria Gabriella Brizi, Marco Cintoni, Pauline Raoul, Palma Maurizi, Giorgio Attinà, Stefano Mastrangelo, Antonio Gasbarrini, Maria Cristina Mele, Antonio Ruggiero

**Affiliations:** 1 UOSD di Oncologia Pediatrica, Dipartimento di Scienze della Salute della Donna, del Bambino e di Sanità Pubblica, Fondazione Policlinico Universitario A. Gemelli IRCCS, Largo A. Gemelli 8, 00168 Rome, Italy; albertoromano90.ar@gmail.com (A.R.); silvia.triarico@guest.policlinicogemelli.it (S.T.); palma.maurizi@unicatt.it (P.M.); giorgio.attina@policlinicogemelli.it (G.A.); stefano.mastrangelo@unicatt.it (S.M.); Antonio.Ruggiero@unicatt.it (A.R.); 2 UOC di Nutrizione Clinica, Dipartimento di Scienze Mediche e Chirurgiche, Fondazione Policlinico Universitario A. Gemelli IRCCS, Largo A. Gemelli 8, 00168 Rome, Italy; marco.cintoni@gmail.com (M.C.); mariacristina.mele@unicatt.it (M.C.M.); 3 Dipartimento di Medicina e Chirurgia Traslazionale, Università Cattolica del Sacro Cuore, Largo F. Vito 1, 00168 Rome, Italy; pauline.raoul1@gmail.com (P.R.); antonio.gasbarrini@unicatt.it (A.G.); 4 Dipartimento di Diagnostica per Immagini, Radioterapia Oncologica ed Ematologia, Fondazione Policlinico Universitario A. Gemelli IRCCS, Largo A. Gemelli 8, 00168 Rome, Italy; luigi.natale@unicatt.it (L.N.); mariagabriella.brizi@policlinicogemelli.it (M.G.B.); 5 Dipartimento di Scienze Radiologiche ed Ematologiche, Università Cattolica del Sacro Cuore, Largo F. Vito 1, 00168 Rome, Italy; 6 Dipartimento di Scienze della Vita e Sanità Pubblica, Università Cattolica del Sacro Cuore, Largo F. Vito 1, 00168 Rome, Italy; 7 UOC di Medicina Interna e Gastroenterologia, Dipartimento di Scienze Mediche e Chirurgiche, Fondazione Policlinico Universitario A. Gemelli IRCCS, Largo A. Gemelli 8, 00168 Rome, Italy

**Keywords:** soft tissue sarcoma, bone sarcoma, pediatric patients, chemotherapy, psoas muscle area (PMA), sarcopenia, personalized medicine

## Abstract

Background: We evaluated nutritional and sarcopenia status and their clinical impact in pediatric patients affected by bone and soft tissue sarcomas. Methods: Body mass index (BMI), prognostic nutritional index (PNI), and total psoas muscle area (tPMA) at diagnosis and after 12 months were analyzed. tPMA was measured from single cross-sectional computed tomography (CT) images at L4–L5. Age-specific and sex-specific tPMA Z-scores were retrieved from an online calculator. Results: A total of 21 patients were identified between February 2013 and December 2018. Twelve patients (57.1%) experienced sarcopenia at diagnosis, although not statistically associated with overall survival (OS) (*p* = 0.09). BMI Z-score, PNI, and tPMA Z-score significantly decreased between diagnosis and after 12 months of treatment (*p* < 0.05). Univariate analysis showed significant associations between poor OS and the presence of metastasis (*p* = 0.008), the absence of surgery (*p* = 0.005), PNI decrease (*p* = 0.027), and the reduction in tPMA > 25% (*p* = 0.042) over the 12 months. Conclusions: Sarcopenia affects more than half of the patients at diagnosis. Decreased PNI during 12 months of treatment has significant predictive value for OS. The role of tPMA derived from CT scan among pediatric patients with sarcoma should be investigated in further prospective and larger studies.

## 1. Introduction

Nutritional status plays a key role in the growth, response to treatment, related complications, quality of life, cost of care, and hospital stay of pediatric patients admitted for various types of illness [1]. In 2013, the Academy of Nutrition and Dietetics of the American Society of Parenteral and Enteral Nutrition (ASPEN) defined pediatric malnutrition status as “an imbalance between nutritional needs and nutrient intake, resulting in a cumulative deficit of energy, protein, or micronutrients that may adversely affect growth, development, and other clinical outcomes” [2].

Pediatric hospital malnutrition is still an underestimated problem, although it results in significant morbidity and mortality among hospitalized children. Several studies have reported a prevalence of 6–51% of this condition among hospitalized pediatric patients [3,4]. The European Society for Clinical Nutrition and Metabolism at Risk (ESPEN) and the European Society for Gastroenterology, Hepatology, and Nutrition (ESPGHAN) recommend screening for nutritional risk in hospitalized children on admission to the ward to facilitate the identification of nutritionally at-risk children and to allow the physician to prepare an appropriate nutritional support plan [5,6,7].

In children hospitalized for cancer, malnutrition constitutes a very common complication, and it is influenced by the type and extent of disease, the intensity of treatment, and the patient’s living conditions [8]. In these patients, the inflammatory response of the underlying disease induces high-energy expenditure and protein catabolism with a net lean body mass loss. In addition, gastrointestinal symptoms such as nausea, vomiting, and lack of appetite (caused by the therapy and/or the tumor) further aggravate the state of protein-energy malnutrition, sometimes exacerbating the toxic effects of therapy and susceptibility to complications [9].

Early identification of the risk and presence of malnutrition, coupled with rapid and personalized nutritional intervention, could, in turn, enhance treatment adherence, reduce treatment complications, and improve patients’ quality of life [10,11,12].

The prognostic nutritional index (PNI) is calculated using the serum albumin concentration and peripheral blood lymphocyte count. PNI has been recognized as a valid indicator of the nutritional and immune status among cancer patients and as an independent prognostic indicator of various malignant tumors [13,14,15,16].

Numerous studies have tried to quantify the effect of malnutrition on the survival of pediatric cancer patients, showing a close relationship between the body mass index (BMI) and mortality [17]. However, the BMI is a nutritional indicator based on the weight and height of the patient, without taking into account the real body composition and the proportions of fat and lean tissue. On the other hand, the loss of muscle tissue and the alteration of body composition may occur as an effect of malnutrition, regardless of weight changes [18]. In pediatric patients with neoplasms, sarcopenia may coexist in the context of malnutrition, amplifying its negative impact on the patients’ prognosis. Sarcopenia is a pathological condition, characterized by a progressive and generalized reduction in the quantity, quality, and strength of muscle mass, more or less associated with reduced physical performance. It is a major cause of physical disability, poor quality of life, loss of self-sufficiency, and death [19,20]. Despite that sarcopenia is generally associated with aging (primary sarcopenia) [21], there are many inflammatory conditions (such as chronic inflammatory diseases and cancer) yielding its occurrence even at a young age as an effect of the catabolic state (secondary sarcopenia) [22]. The development of sarcopenia in adult cancer patients is closely linked to an increase in mortality and morbidity, independently from the BMI variation [23,24]. In the pediatric age, the presence of sarcopenia has been linked to the mortality and morbidity of numerous chronic diseases and in patients with aggressive and disabling malignant pathologies, even if the evidence is still scarce [25,26].

There are several ways to assess muscle mass and possible sarcopenia status in clinical practice [27]. In adults, the skeletal muscle mass index (SMI) is calculated from the skeletal muscle area (SMA) (cm^2^) divided by the square of the patient’s height (m^2^) [28,29,30]. SMI is indicative of sarcopenia if less than 55 cm^2^/m^2^ in males and 39 cm^2^/m^2^ in females [31,32]. This method has been widely adopted for the analysis of muscle mass in adult patients with neoplasia, as well as in patients with liver disease, patients in intensive care units, and patients undergoing surgery [33,34,35,36]. Recently, Lurz et al. generated age- and sex-specific curves related to total psoas muscle area (tPMA) derived at L3–L4 or L4–L5, which can be used for pediatric patients aged 1 to 16 years. From them, the Z-scores of the PMA can be derived through a calculator available online (https://ahrc-apps.shinyapps.io/sarcopenia/ (accessed on 15 December 2021)) and easily identify the presence of sarcopenia [37]. Although Lurz et al. analyzed tPMA at both L3–L4 and L4–L5 levels, tPMA at L4–L5 seems to be more relevant in the pediatric age group, because at this level the psoas muscle shape is also rounder than at L3–L4, allowing for more accurate contour drawing. In addition, L4–L5 is the reference level for the assessment of visceral adipose tissue, so an analysis at this level is a reliable measure of both skeletal muscle and adipose tissue [38,39]. Bone and soft tissue sarcomas of childhood are highly aggressive pathologies, which require intensive chemotherapy treatments and can be burdened by a high rate of immobilization resulting from surgical procedures or pain. Prolonged immobilization further causes a reduction in muscle mass and strength, with a consequent possible worsening of the state of sarcopenia [40].

In the present study, we evaluate body composition changes, sarcopenia assessed by the evaluation of PMA obtained from computed tomography (CT) scans, and their impact on the survival and prognosis of pediatric patients with bone and soft tissue sarcomas.

## 2. Materials and Methods

### 2.1. Study Endpoints

The primary endpoint was:

- To describe the clinical characteristics, nutritional status (defined by the BMI and the PNI), and the presence of sarcopenia (defined by the measurement of tPMA detected by axial CT images of the L4–L5 vertebrae) at diagnosis and after 12 months of chemotherapy in pediatric patients with bone and soft tissue sarcomas.

The secondary endpoints were:

- To evaluate the association between clinical characteristics, nutritional status, and the presence of sarcopenia (at diagnosis) with overall survival (OS);
- To evaluate the association between the presence of sarcopenia (at diagnosis) with any infectious complications due to treatment (defined as the number of hospitalizations for febrile neutropenia in the 12 months of observation).

### 2.2. Study Design, Patients’ Characteristics, Ethical Approval, Inclusion, and Exclusion Criteria

In this observational pilot study, data from twenty-two pediatric patients with a new diagnosis of bone and soft tissue sarcoma, admitted at the Pediatric Oncology Unit of the Fondazione Policlinico Agostino Gemelli IRCCS from February 2013 to December 2018, were retrospectively analyzed.

The ethical approval was obtained from the Ethics Committee of our institution (Protocol ID 4211, Approval Letter Number 36155/21). Informed consent was obtained from the parents or the legal guardians of the enrolled patients. The study was carried out following the Helsinki Declaration of Human Rights.

Inclusion criteria were: diagnosis of bone or soft tissue sarcomas (Ewing sarcoma, rhabdomyosarcoma, desmoplastic tumor), age between 1 and 16 years, and staging of the disease by CT scan of the abdomen at diagnosis and after 12 months of treatment.

The exclusion criterion was the absence of CT images available at the time of data analysis.

### 2.3. Clinical Data and Nutritional Assessment

For each patient, the following data were collected:

- Demographic characteristics (age, sex, ethnicity);
- Data related to the neoplastic disease (histology, primarily affected site, date of diagnosis, and presence of metastasis at diagnosis);
- Type and duration of the treatment (chemotherapy protocol, radiotherapy, and high-dose chemotherapy with autologous transplantation);
- Surgery during the 12 months of observation;
- Five-year OS defined as the time from the day of diagnosis to death from any cause in five years after diagnosis;
- Data on treatment-related infectious complications (febrile neutropenia that required hospitalization and intravenous antibiotic therapy) in the 12 months of observation.

For each patient, the assessment of nutritional status was performed according to the following variables: BMI, BMI Z-score, PNI, and tPMA at diagnosis and after 12 months.

The BMI was calculated with the formula: weight (kg)/height (m^2^). The BMI Z-score was calculated with “PediTools-Clinical tools for pediatric providers”, available on the internet (https://peditools.org/, last (accessed on 20 September 2021)). The change in BMI (ΔBMI) over the 12 months was calculated with the formula: ((BMI after 12 months—BMI at diagnosis)/(BMI at diagnosis)) × 100.

The PNI, as an indicator of the nutritional and inflammatory status, was calculated according to the formula: (10 × serum albumin (g/dL)) + (0.005 × number of lymphocytes/μL). The change in the PNI (ΔPNI) over the 12 months was calculated with the formula: ((PNI after 12 months − PNI at diagnosis)/(PNI at diagnosis)) × 100.

To evaluate the presence of sarcopenia, tPMA was measured from single cross-sectional abdominal CT images at the level of L4–L5 at diagnosis and after 12 months of treatment. The tPMA expressed in mm^2^ was obtained using the SliceOmatic software v5.0 (Tomovision, Montreal, QC, Canada) and was measured at the level of L4–L5 because most clinically relevant and useful according to Lurz et al. [37]. A calculator available online (https://ahrc-apps.shinyapps.io/sarcopenia/) (accessed on 15 December 2021) was used to obtain the tPMA Z-scores in relation to sex and age of the patient. The degree of sarcopenia was defined by the following parameters: mild for <−1 Z-score, moderate for <−2 Z-score, severe for <−3 Z-score. The change in tPMA (ΔtPMA) over the 12 months was calculated with the formula: ((tPMA after 12 months—tPMA at diagnosis)/(tPMA at diagnosis)) × 100.

### 2.4. Sample Size and Statistical Analysis

Given the lack of data on the presence and degree of sarcopenia in the population of interest, we set the sample size for this pilot study at *n* = 22 patients. This number intercepts various expected proportions, with a confidence level of 95% and a margin of error ranging from a minimum of 4.16% (for an expected proportion of 1%) to a maximum of 20.89 (for an expected proportion of 50%).

Continuous data are given as median and interquartile range (IQR: 25–75° percentile) and were compared using Mann–Whitney U tests. Categorical data are presented as absolute and percentage frequencies and were compared using the Chi-squared test or Fisher Exact Test, where appropriate. The study of the correlations was carried out using Pearson’s test.

OS was estimated by the Kaplan–Meier method and was tested with the log-rank test. OS was defined as the time between diagnosis and death, expressed in months. A Cox regression proportional hazards model was constructed to assess the prognostic significance of covariates. The hazard ratio (HR) with 95% CI was reported as an estimate of the risk of death. All statistical analyses were performed using XLSTAT version 2021.3.1 by Addinsoft. Values of *p* < 0.05 were considered statistically significant.

## 3. Results

### 3.1. Clinical Characteristics, Nutritional Status, Sarcopenia at Diagnosis and 12 Months

A total of 22 patients diagnosed with a bone or soft tissue sarcoma and treated at our center were included between February 2013 and December 2018. Only one patient was excluded, due to the impossibility of obtaining the CT images at12 months after diagnosis.

Demographic and clinical characteristics of the enrolled population are reported in Table 1.

Eleven (52.4%) patients were male, and ten (47.6%) were female, with a median age of 125.6 (78.7; 181.5) months. There were 14 (66.6%) diagnoses of Ewing sarcoma, 6 (28.6%) of rhabdomyosarcoma, and 1 (4.8%) of desmoplastic tumor.

At diagnosis, no patient could undergo complete resection of the primitive mass. As per EpSSG RMS 05 protocol, all patients with rhabdomyosarcoma or desmoplastic tumor who did not have metastases at diagnosis and who could not undergo complete resection of the mass were classified as stage III, while those with metastases were classified as stage IV. Similarly, all patients with Ewing’s sarcoma without metastases at diagnosis and who could not undergo complete resection of the mass were classified as stage III, and those with metastases were classified as stage IV.

At diagnosis, the median of the BMI Z-score was −0.23 (−0.89; 0.85), the median of PNI was 48.1 (45.2; 53.2), and the median of the tPMA Z-score was −1.01 (−1.71; −0.35). At diagnosis, 12 (57.2%) were sarcopenic, 8 (38.1%) mild, and 4 (19.1%) moderate sarcopenic. None were diagnosed with severe sarcopenia.

Patients with Ewing sarcoma were treated according to the EURO-EWING99 protocol: 4 (28.6%) of them received high-dose chemotherapy with busulfan and melphalan, followed by autologous stem cell transplantation, 3 (21.4%) underwent radiotherapy, and 13 (92.9%) underwent surgical excision of the primary residual mass. Patients with rhabdomyosarcoma and desmoplastic tumor were treated according to the EpSSG RMS 05 protocol. None received high-dose chemotherapy or radiotherapy. One patient with rhabdomyosarcoma and desmoplastic tumor required primary tumor surgery, followed by chemotherapy according to the EpSSG NRSTS 05 protocol.

At the time of the analysis, 11 patients (52.4%) were dead, and 13 patients (61.9%) had disease progression.

Table 2 shows the comparison of nutritional variables at diagnosis and 12 months after the beginning of treatment. The differences in terms of presence of sarcopenia at diagnosis and after 12 months of treatment were not statistically significant, whereas significant differences were observed for the BMI Z-score, the PNI, and the tPMA Z-score.

**Figure 1:**
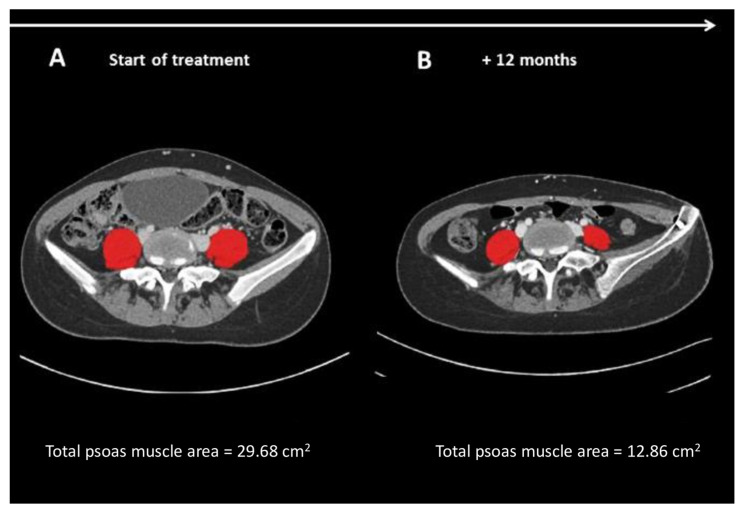
tPMA (in red color) measured from single cross-sectional abdominal CT images at the level of L4–L5 in the same patient, at diagnosis, and after 12 months of treatment.

Figure 1 represents the change of tPMA, measured from single cross-sectional abdominal CT images at the level of L4–L5 in the same patient at diagnosis and after 12 months of treatment.

### 3.2. Association between Clinical Characteristics, Nutritional Status, Sarcopenia, and Overall Survival

Among the sarcopenic patients at diagnosis, seven (58.3%) were affected by Ewing sarcoma and five (41.7%) by rhabdomyosarcoma (*p* = 0.16).

Table 3 describes the potential associations between clinical and nutritional variables and OS, as a result of Cox proportional hazards analysis. The presence of mild or moderate sarcopenia at diagnosis was not statistically associated with OS (HR = 3.19 (0–82–12.36); *p* = 0.09). In the univariate analysis, the presence of metastasis, the absence of surgery during the 12 months, the ΔPNI, and a reduction of PMA greater than 25% over 12 months were significantly associated with poor OS.

### 3.3. Association between Sarcopenia and Infectious Complications

**Figure 2:**
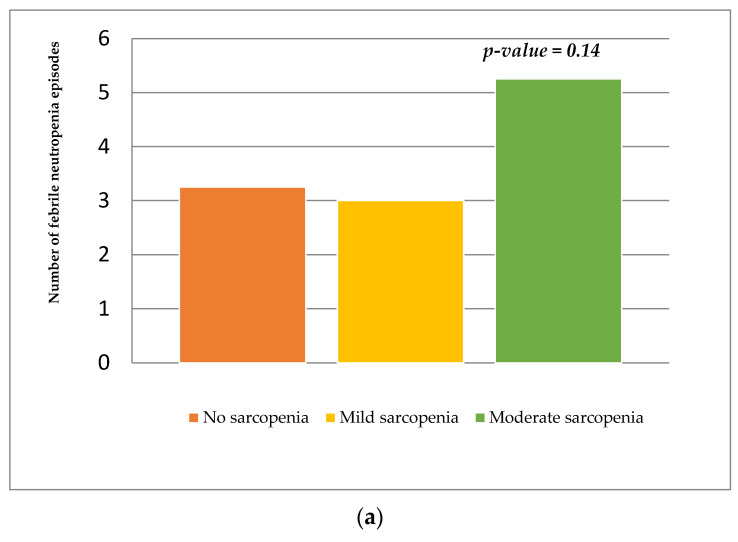

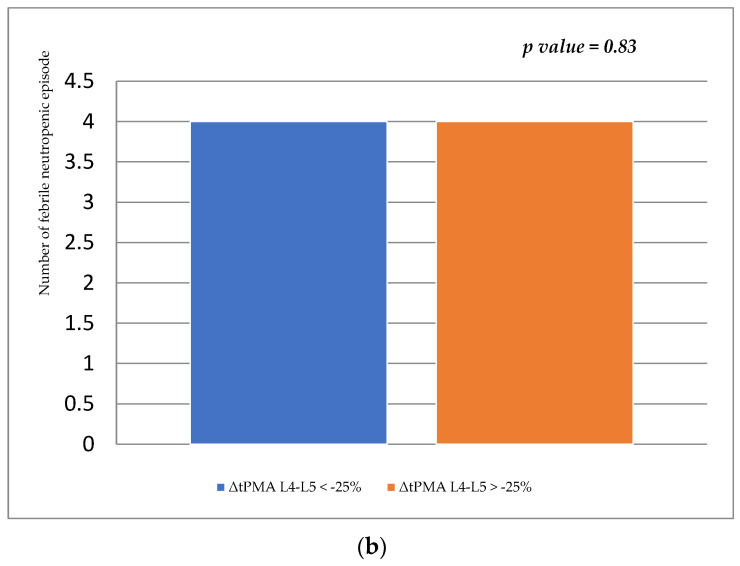
(**a**) Number of febrile neutropenic episodes in nonsarcopenic, mildly sarcopenic, and moderately sarcopenic patients at diagnosis. (**b**) Number of febrile neutropenic episodes in the patients with ΔtPMA L4–L5 < −25% and patients with ΔtPMA L4–L5 > −25%.

The median number of febrile neutropenic episodes that require hospitalization and intravenous antibiotic therapy was 3 (IQR = 3–7) in nonsarcopenic patients, 2.5 (IQR = 0.5–4.5) in mild sarcopenic patients, and 5 (IRQ 1–5.5) in moderate sarcopenic patients. The comparison between the median number of febrile neutropenic episodes that require hospitalization and intravenous antibiotic therapy was not statistically different among the nonsarcopenic, mild sarcopenic, and moderate sarcopenic groups at diagnosis, as shown in Figure 2a. Additionally, the median number of febrile neutropenic episodes that require hospitalization and intravenous antibiotic therapy was 2 (IQR = 2–5.75) in patients with ΔtPMA L4–L5 < −25% and 2 (IQR = 3.5–5) in patients with ΔtPMA L4–L5 > −25%. The comparison between the median number of febrile neutropenic episodes that require hospitalization and intravenous antibiotic therapy was not statistically different among the patients with ΔtPMA L4–L5 < −25% and patients with ΔtPMA L4–L5 > −25%, as shown in Figure 2b. The study of correlations using Pearson’s test showed that there was no association between Δ tPMA L4–L5 and the number of febrile neutropenic episodes (*r* = −0.25; *p* = 0.06) and between ΔBMI and the number of febrile neutropenic episodes (*r* = −0.3; *p* = 0.1). A weak association was observed between ΔPNI and the number of febrile neutropenic episodes (*r* = 0.1; *p* = 0.01).

## 4. Discussion

This is a pilot retrospective study investigating sarcopenia and nutritional status at diagnosis among inpatient children affected by cancer and their potential clinical impact. The recently published study of Lurz et al. defining pediatric reference curves for tPMA allowed us to diagnose sarcopenia in children using CT scan [37]. Indeed, by using the reference curves we observed that sarcopenia in children with cancer at diagnosis is a greatly more widespread condition than we might have expected. We can speculate that the cause of this phenomenon could be found in the pathogenetic mechanisms of sarcopenia. Secondary sarcopenia is caused by the coexistence of multiple pathogenic mechanisms involving the increase in apoptotic activity in muscle cells, the production of inflammatory cytokines such as tumor necrosis factor (TNF)-alpha and interleukin (IL)-6, the presence of oxidative stress, and accumulation of oxygen radicals, low energy and protein intake [41]. Even before the tumor diagnosis, it is possible that these mechanisms, especially the neoplastic, inflammatory, microenvironmental (TNF, IL-1, proteolysis-inducing factor), and physical inactivity [42], play a role in the pathogenesis of sarcopenia. In our opinion, such a high rate of sarcopenia at diagnosis in children with soft tissue and bone sarcomas could also be related to the diagnostic delay of these tumors [43].

By comparing the mean value of tPMA at diagnosis and after 12 months, we observed a statistically significant reduction in the tPMA Z-score. This change during treatment may probably be caused by an inadequate intake of energy or proteins (due to anorexia, nausea, vomiting, malabsorption related to the oncologic treatments), already associated with the inflammatory state and hypomobility [42]. A reduced intake of nutrients during cancer treatment can be associated with sarcopenia diagnosis or can worsen its severity if already present before initiating treatment. As observed by Chindapasirt et al., sarcopenia may produce a vicious circle, causing the worsening of the patient’s inflammatory status [44]. In our population, this resulted in the fact that after 12 months of treatment only 23% of the patients did not develop sarcopenia. A previous report also showed an increased rate of pediatric sarcopenia status during treatment of acute lymphoblastic leukemia, but the authors assessed the skeletal muscle mass using dual-energy X-ray absorptiometry scans without exactly defining sarcopenia status at diagnosis [45]. In our study, the incidence of sarcopenia in children with cancer and its variations during treatment have been precisely defined from the analysis of CT scan routinely used at diagnosis for the staging of sarcomas. This means that the diagnosis of sarcopenia was easily made without the need for supplementary procedures and additional costs.

Furthermore, BMI Z-score and PNI significantly decreased from diagnosis to 12 months after treatment. The mechanisms underlying the reduction of BMI Z-score and PNI could be similar to those of the reduction in muscle mass in children with cancer. PNI is a nutritional and inflammatory marker associated with survival in numerous studies of cancer patients. It is calculated on the basis of albumin values, which are compromised both by the inflammatory status and the reduced protein intake and by lymphocytes, whose values may also be affected by myelotoxic chemotherapy [14].

The univariate analysis showed that metastatic disease, the absence of surgery during the 12 months, the ΔPNI and ΔtPMA L4–L5 > −25% adversely affected the prognosis of the population studied. This has not been shown for the change in BMI. Indeed, BMI is not a marker of muscle mass, especially in inflammatory conditions. The decline in visceral and muscle proteins generally occurs with an increase in extracellular volume, so body weight and BMI may vary little until cachexia becomes evident [46]. It has also been observed that pediatric patients with poor prognosis frequently have a high BMI due to an excess of fat or edema. In recent decades, sarcopenic obesity has emerged as a pathological condition with a loss of muscle tissue associated with an increase in adipose tissue and body weight [47]. The presence of sarcopenic obesity may adversely affect the prognosis of adult patients with cancer [47,48]. The lack of significance of the variation of the BMI Z-score as a negative prognostic factor in our population is probably the result of this mechanism.

As we have observed in children with soft tissue and bone sarcomas, a relationship between tPMA and survival has also been demonstrated in children with hepatoblastoma and neuroblastoma [49,50]. Ritz et al. observed that tPMA in children with hepatoblastoma was significantly lower than in healthy controls, even in the presence of normal body weight and height. Moreover, children with tPMA under the fifth percentile showed an increased risk of relapse and liver transplant [49]. Finally, Kawakubo et al. analyzed the rate of change in the tPMA of L3 level on CT images before and after treatment in fifteen children with high-risk neuroblastoma. They found shorter progression-free survival (PFS) and OS in the group of patients who presented a reduced skeletal muscle mass during the treatment [50].

Unlike the study conducted by Rayar et al. in children with acute lymphoblastic leukemia, the presence of sarcopenia at diagnosis and the reduction of tPMA during treatment seem not to influence the number of infectious episodes recorded during the observation [45]. However, Rayar et al. conducted their studies among children with leukemia during the induction treatment when infections are very frequent due to the intensity of the treatment to leukemia itself, which compromises the immune system [45]. Moreover, during treatment, children with leukemia undergo a modification of the gut microbiome, which can affect the nutritional status of the patients, causing an increase in infectious episodes [51]. In soft tissue and bone sarcomas, the treatment has a decisively lower myeloablative effect compared to the induction phase of leukemia with a consequent lower incidence of infectious episodes.

Our results failed to show a significant reduction in skeletal muscle mass during treatment as a prognostic value. It could be due to the small sample size of the population studied, representing the main limitations of this pilot study. However, we showed a significant reduction in tPMA between diagnosis and 12 months after treatment at an age in which muscle mass and weight should physiologically increase. Moreover, this is the first study that used tPMA measurement in the clinical setting as a marker of sarcopenia in childhood cancer. We acknowledge that tPMA is easy to use and can be determined through a routinely abdominal CT. Since all patients with soft tissue and bone sarcoma undergo numerous disease stages with abdominal CT, tPMA can be simply performed also during the treatment and the follow-up visits. Further studies with a more robust design and higher sample size are warranted to confirm or rebut our results.

## 5. Conclusions

Sarcopenia may occur in pediatric patients with soft tissue and bone sarcoma already at diagnosis. The diagnosis of sarcopenia can be easily performed by measuring the tPMA from single cross-sectional abdominal CT images at the level of L4–L5, according to tPMA Z-scores specific for the sex and age of the patients. Our study showed a reduction of tPMA Z-scores between diagnosis and at 12 months of treatment. This could be crucial for OS, tolerance, and the response to the treatment; therefore, careful surveillance should be performed, to allow the implementation of nutritional and physical rehabilitation measures. Furthermore, significant differences in BMI Z-scores and PNI may be observed after 12 months of treatment, and consequently, nutritional and immune status should be closely monitored throughout the treatment.

Prospective and larger studies may help to better investigate the role of tPMA derived from CT scan in this particular setting of patients, to implement their use in the clinical practice, allowing a therapeutic approach as personalized as possible.

## Figures and Tables

**Table 1:** Clinical characteristics, nutritional status, and sarcopenia at diagnosis (*n* = 21).

Characteristics	Number of Patients (%) or Median IQR
Age (months)	125.6 (78.7; 181.5)
Sex (male)	11 (52.4)
Histology	
Ewing sarcoma	14 (66.6)
Rhabdomyosarcoma	6 (28.6)
Desmoplastic tumor	1 (4.8)
Primary localization	
Bone	13 (61.9)
Soft tissue	8 (38.1)
Presence of metastases at diagnosis	9 (42.9)
BMI (kg/m^2^)	17.4 (15; 19.6)
BMI (percentile)	41.7 (0.9–86.2)
BMI Z-Score	−0.23 (−0.89; 0.85)
PNI	48.1 (45.2; 53.2)
tPMA L4–L5 (mm^2^)	13.21 (8.79; 19.38)
tPMA L4–L5 Z-Score	−1.01 (−1.71; −0.35)
Nonsarcopenic patients	9 (42.8)
Sarcopenic patients	12 (57.1)
Mild	8 (38.1)
Moderate	4 (19.1)
Severe	0 (0)
Progressive disease	13 (61.9)
Deaths	11 (51.4)

Abbreviations: tPMA, total area of the psoas muscle; BMI, body mass index; IQR, interquartile range; PNI, prognostic nutritional index.

**Table 2:** Comparison of nutritional status variables and sarcopenia status rates at diagnosis and after 12 months of treatment.

Variables	Diagnosis (Median (25°; 75°) or Number of Patients (%))	12 Months (Median (25°; 75°) or Number of Patients (%))	*p* Value
BMI (kg/m^2^)	17.4 (15; 19.6)	17.0 (13.6; 19.2)	0.25
BMI (percentile)	41.7 (0.9–86.2)	12.2 (3.9–72.9)	0.28
BMI Z-score	−0.23 (−0.89; 0.85)	−1.17 (−2.11; 0.48)	0.01
PNI	48.1 (45.2; 53.2)	39.6 (35.7; 45.6)	0.001
tPMA L4–L5 (mm^2^)	13.2 (8.8; 19.4)	11.5 (9.95; 18.48)	0.31
tPMA L4–L5 Z-score	−1.01 (−1.71; −0.35)	−1.46 (−2.57; −1.11)	0.005
Nonsarcopenic patients	9 (42.8)	5 (23.8)	0.32
Sarcopenic patients			
Mild	8 (38.1)	8 (38.1)	0.999
Moderate	4 (19.1)	8 (38.1)	0.29
Severe	0 (0)	0 (0)	-

Abbreviations: tPMA: total area of the psoas muscle; BMI: body mass index; PNI prognostic nutritional index.

**Table 3:** Univariate analysis of clinical characteristics, nutritional status, and sarcopenia for overall survival.

	HR (95%CI)	*p* Value
Absence of sarcopenia at diagnosis	0.63 (0.18–2.15)	0.461
Mild sarcopenia at diagnosis	0.8 (0.24–2.75)	0.726
Moderate sarcopenia at diagnosis	3.19 (0.82–12.36)	0.09
Ewing sarcoma	0.35 (0.1–1.15)	0.08
Rhabdomyosarcoma	2.86 (0.86–9.4)	0.08
Presence of metastases at diagnosis	6.07 (1.59–23)	0.008
High-dose chemotherapy	0.39 (0.05–3.13)	0.38
Radiotherapy	0.49 (0.06–3.83)	0.49
Absence of surgery	0.17 (0–0.6)	0.005
BMI at diagnosis	1.02 (0.96–1.07)	0.52
PNI at diagnosis	1 (0.95–1.07)	0.76
tPMA L4–L5 Z-score at diagnosis	1 (0.52–2)	0.95
ΔBMI	1.01 (0.97–1.01)	0.59
ΔPNI	0.966 (0–0.99)	0.027
ΔtPMA L4–L5	0.97 (0.94–1)	0.122
ΔtPMA L4–L5 > −25%	4.14 (1.05–16.3)	0.042

Abbreviations: tPMA: total area of the psoas muscle; BMI: body mass index; PNI prognostic nutritional index; ΔBMI (the change of BMI over the 12 months of treatment); ΔPNI (the change of PNI over the 12 months of treatment); ΔtPMA (the change of PMA over the 12 months); ΔtPMA L4–L5 > −25% (reduction of PMA greater than 25% over 12 months).

## Data Availability

The data presented in this study are available on request from the corresponding author for any academic use upon citation of this article. The data are not publicly available due to privacy and permission restricted to the publication of this article only.

## References

[1] Mehta N.M., Corkins M.R., Lyman B., Malone A., Goday P.S., Carney L.N., Monczka J.L., Plogsted S.W., Schwenk W.F. (2013). Defining pediatric malnutrition: A paradigm shift toward etiology-related definitions. JPEN J. Parenter. Enteral Nutr..

[2] Beer S.S., Juarez M.D., Vega M.W., Canada N.L. (2015). Pediatric malnutrition: Putting the new definition and standards into practice. Nutr. Clin. Pract..

[3] Joosten K.F., Hulst J.M. (2008). Prevalence of malnutrition in pediatric hospital patients. Curr. Opin. Pediatr..

[4] Hartman C., Shamir R., Hecht C., Koletzko B. (2012). Malnutrition screening tools for hospitalized children. Curr. Opin. Clin. Nutr. Metab. Care.

[5] Agostoni C., Axelson I., Colomb V., Goulet O., Koletzko B., Michaelsen K.F., Puntis J.W., Rigo J., Shamir R., Szajewska H. (2005). ESPGHAN Committee on Nutrition; European Society for Paediatric Gastroenterology. The need for nutrition support teams in pediatric units: A commentary by the ESPGHAN committee on nutrition. J. Pediatr. Gastroenterol. Nutr..

[6] Teixeira A.F., Viana K.D. (2016). Nutritional screening in hospitalized pediatric patients: A systematic review. J. Pediatr..

[7] Rinninella E., Ruggiero A., Maurizi P., Triarico S., Cintoni M., Mele M.C. (2017). Clinical tools to assess nutritional risk and malnutrition in hospitalized children and adolescents. Eur. Rev. Med. Pharmacol. Sci..

[8] Iniesta R.R., Paciarotti I., Brougham M.F., McKenzie J.M., Wilson D.C. (2015). Effects of pediatric cancer and its treatment on nutritional status: A systematic review. Nutr. Rev..

[9] Zimmermann K., Ammann R.A., Kuehni C.E., De Geest S., Cignacco E. (2013). Malnutrition in pediatric patients with cancer at diagnosis and throughout therapy: A multicenter cohort study. Pediatr. Blood Cancer.

[10] Loeffen E.A., Brinksma A., Miedema K.G., de Bock G.H., Tissing W.J. (2015). Clinical implications of malnutrition in childhood cancer patients--infections and mortality. Support. Care Cancer.

[11] Joffe L., Schadler K.L., Shen W., Ladas E.J. (2019). Body Composition in Pediatric Solid Tumors: State of the Science and Future Directions. J. Natl. Cancer Inst. Monogr..

[12] Triarico S., Rinninella E., Cintoni M., Capozza M.A., Mastrangelo S., Mele M.C., Ruggiero A. (2019). Impact of malnutrition on survival and infections among pediatric patients with cancer: A retrospective study. Eur. Rev. Med. Pharmacol. Sci..

[13] Ikeya T., Shibutani M., Maeda K., Sugano K., Nagahara H., Ohtani H., Hirakawa K. (2015). Maintenance of the nutritional prognostic index predicts survival in patients with unresectable metastatic colorectal cancer. J. Cancer Res. Clin. Oncol..

[14] Hofbauer S.L., Pantuck A.J., de Martino M., Lucca I., Haitel A., Shariat S.F., Belldegrun A.S., Klatte T. (2015). The preoperative prognostic nutritional index is an independent predictor of survival in patients with renal cell carcinoma. Urol. Oncol..

[15] Miao J., Xiao W., Wang L., Han F., Wu H., Deng X., Guo X., Zhao C. (2017). The value of the Prognostic Nutritional Index (PNI) in predicting outcomes and guiding the treatment strategy of nasopharyngeal carcinoma (NPC) patients receiving intensity-modulated radiotherapy (IMRT) with or without chemotherapy. J. Cancer Res. Clin. Oncol..

[16] Zhang H., Shang X., Ren P., Gong L., Ahmed A., Ma Z., Wu X., Xiao X., Jiang H., Tang P. (2019). The predictive value of a preoperative systemic immune-inflammation index and prognostic nutritional index in patients with esophageal squamous cell carcinoma. J. Cell Physiol..

[17] Ooi P.H., Thompson-Hodgetts S., Pritchard-Wiart L., Gilmour S.M., Mager D.R. (2020). Pediatric Sarcopenia: A Paradigm in the Overall Definition of Malnutrition in Children?. JPEN J. Parenter. Enteral Nutr..

[18] Landi F., Calvani R., Cesari M., Tosato M., Martone A.M., Ortolani E., Savera G., Salini S., Sisto A., Picca A. (2018). Sarcopenia: An Overview on Current Definitions, Diagnosis and Treatment. Curr. Protein Pept. Sci..

[19] Cruz-Jentoft A.J., Baeyens J.P., Bauer J.M., Boirie Y., Cederholm T., Landi F., Martin F.C., Michel J.P., Rolland Y., Schneider S.M. (2010). European Working Group on Sarcopenia in Older People. Sarcopenia: European consensus on definition and diagnosis: Report of the European Working Group on Sarcopenia in Older People. Age Ageing.

[20] Cruz-Jentoft A.J., Bahat G., Bauer J., Boirie Y., Bruyère O., Cederholm T., Cooper C., Landi F., Rolland Y., Sayer A.A. (2019). Writing Group for the European Working Group on Sarcopenia in Older People 2 (EWGSOP2), and the Extended Group for EWGSOP2. Sarcopenia: Revised European consensus on definition and diagnosis. Age Ageing.

[21] Mitchell W.K., Williams J., Atherton P., Larvin M., Lund J., Narici M. (2012). Sarcopenia, dynapenia, and the impact of advancing age on human skeletal muscle size and strength; a quantitative review. Front. Physiol..

[22] Marhold M., Topakian T., Unseld M. (2021). Sarcopenia in cancer—a focus on elderly cancer patients. memo-Mag. Eur. Med. Oncol..

[23] Martin L., Birdsell L., Macdonald N., Reiman T., Clandinin M.T., McCargar L.J., Murphy R., Ghosh S., Sawyer M.B., Baracos V.E. (2013). Cancer cachexia in the age of obesity: Skeletal muscle depletion is a powerful prognostic factor, independent of body mass index. J. Clin. Oncol..

[24] Lai H.J. (2006). Classification of nutritional status in cystic fibrosis. Curr. Opin. Pulm. Med..

[25] Kim S., Koh H. (2015). Nutritional aspect of pediatric inflammatory bowel disease: Its clinical importance. Korean J. Pediatr..

[26] Carvalho do Nascimento P.R., Poitras S., Bilodeau M. (2018). How do we define and measure sarcopenia? Protocol for a systematic review. Syst. Rev..

[27] Abd Aziz N.A.S., Teng N., Abdul Hamid M.R., Ismail N.H. (2017). Assessing the nutritional status of hospitalized elderly. Clin. Interv. Aging.

[28] Orsso C.E., Tibaes J.R.B., Oliveira C.L.P., Rubin D.A., Field C.J., Heymsfield S.B., Prado C.M., Haqq A.M. (2019). Low muscle mass and strength in pediatric patients: Why should we care?. Clin. Nutr..

[29] Cooper C., Fielding R., Visser M., van Loon L.J., Rolland Y., Orwoll E., Reid K., Boonen S., Dere W., Epstein S. (2013). Tools in the assessment of sarcopenia. Calcif. Tissue Int..

[30] Anjanappa M., Corden M., Green A., Roberts D., Hoskin P., McWilliam A., Choudhury A. (2020). Sarcopenia in cancer: Risking more than muscle loss. Tech. Innov. Patient Support Radiat. Oncol..

[31] Amini B., Boyle S.P., Boutin R.D., Lenchik L. (2019). Approaches to assessment of muscle mass and myosteatosis on computed tomography (CT): A systematic review. J. Gerontol. A Biol. Sci. Med. Sci..

[32] Moisey L.L., Mourtzakis M., Cotton B.A., Premji T., Heyland D.K., Wade C.E., Bulger E., Kozar R.A. (2013). Nutrition and Rehabilitation Investigators Consortium (NUTRIC). Skeletal muscle predicts ventilator-free days, ICU-free days, and mortality in elderly ICU patients. Crit. Care.

[33] Akahoshi T., Yasuda M., Momii K., Kubota K., Shono Y., Kaku N., Tokuda K., Nagata T., Yoshizumi T., Shirabe K. (2016). Sarcopenia is a predictive factor for prolonged intensive care unit stays in high-energy blunt trauma patients. Acute Med. Surg..

[34] Yeh D.D., Ortiz-Reyes L.A., Quraishi S.A., Chokengarmwong N., Avery L., Kaafarani H.M.A., Lee J., Fagenholz P., Chang Y., DeMoya M. (2018). Early nutritional inadequacy is associated with psoas muscle deterioration and worse clinical outcomes in critically ill surgical patients. J. Crit. Care.

[35] Giusto M., Lattanzi B., Albanese C., Galtieri A., Farcomeni A., Giannelli V., Lucidi C., Di Martino M., Catalano C., Merli M. (2015). Sarcopenia in liver cirrhosis: The role of computed tomography scan for the assessment of muscle mass compared with dual-energy X-ray absorptiometry and anthropometry. Eur. J. Gastroenterol. Hepatol..

[36] Van Vugt J.L.A., Levolger S., de Bruin R.W.F., van Rosmalen J., Metselaar H.J., Ijzermans J.N.M. (2016). Systematic review and meta-analysis of the impact of computed tomography-assessed skeletal muscle mass on outcome in patients awaiting or undergoing liver transplantation. Am. J. Transplant..

[37] Lurz E., Patel H., Lebovic G., Quammie C., Woolfson J.P., Perez M., Ricciuto A., Wales P.W., Kamath B.M., Chavhan G.B. (2020). Paediatric reference values for total psoas muscle area. J. Cachexia Sarcopenia Muscle.

[38] Morrell G.R., Ikizler T.A., Chen X., Heilbrun M.E., Wei G., Boucher R., Beddhu S. (2016). Psoas Muscle Cross-sectional Area as a Measure of Whole-body Lean Muscle Mass in Maintenance Hemodialysis Patients. J. Ren. Nutr..

[39] Triarico S., Rinninella E., Mele M.C., Cintoni M., Attinà G., Ruggiero A. (2021). Prognostic impact of sarcopenia in children with cancer: A focus on the psoas muscle area (PMA) imaging in the clinical practice. Eur. J. Clin. Nutr..

[40] Rommersbach N., Wirth R., Lueg G., Klimek C., Schnatmann M., Liermann D., Janssen G., Müller M.J., Pourhassan M. (2020). The impact of disease-related immobilization on thigh muscle mass and strength in older hospitalized patients. BMC Geriatr..

[41] Joseph C., Kenny A.M., Taxel P., Lorenzo J.A., Duque G., Kuchel G.A. (2005). Role of endocrine-immune dysregulation in osteoporosis, sarcopenia, frailty and fracture risk. Mol. Aspects Med..

[42] Di Giorgio A., Rotolo S., Cintoni M., Rinninella E., Pulcini G., Schena C.A., Ferracci F., Grassi F., Raoul P., Moroni R. (2021). The prognostic value of skeletal muscle index on clinical and survival outcomes after cytoreduction and HIPEC for peritoneal metastases from colorectal cancer: A systematic review and meta-analysis. Eur. J. Surg. Oncol..

[43] Goedhart L.M., Gerbers J.G., Ploegmakers J.J., Jutte P.C. (2016). Delay in Diagnosis and Its Effect on Clinical Outcome in High-grade Sarcoma of Bone: A Referral Oncological Centre Study. Orthop. Surg..

[44] Chindapasirt J. (2015). Sarcopenia in Cancer Patients. Asian Pac. J. Cancer Prev..

[45] Rayar M., Webber C.E., Nayiager T., Sala A., Barr R.D. (2013). Sarcopenia in children with acute lymphoblastic leukemia. J. Pediatr. Hematol. Oncol..

[46] Murphy A.J., White M., Davies P.S. (2010). Body composition of children with cancer. Am. J. Clin. Nutr..

[47] Polyzos S.A., Margioris A.N. (2018). Sarcopenic obesity. Hormones.

[48] Baracos V.E., Arribas L. (2018). Sarcopenic obesity: Hidden muscle wasting and its impact for survival and complications of cancer therapy. Ann. Oncol..

[49] Ritz A., Kolorz J., Hubertus J., Ley-Zaporozhan J., von Schweinitz D., Koletzko S., Häberle B., Schmid I., Kappler R., Berger M. (2021). Sarcopenia is a prognostic outcome marker in children with high-risk hepatoblastoma. Pediatr. Blood Cancer.

[50] Kawakubo N., Kinoshita Y., Souzaki R., Koga Y., Oba U., Ohga S., Taguchi T. (2019). The Influence of Sarcopenia on High-Risk Neuroblastoma. J. Surg. Res..

[51] Masetti R., Muratore E., Leardini D., Zama D., Turroni S., Brigidi P., Esposito S., Pession A. (2021). Gut microbiome in pediatric acute leukemia: From predisposition to cure. Blood Adv..

